# Temporal Dynamics of Polyp Regression and Retreatment Burden in As-Needed VEGF Inhibitor Therapy: A 24-Month Study

**DOI:** 10.3390/ph19091380

**Published:** 2026-09-01

**Authors:** Misa Kimura, Yoichi Sakurada, Yoshiko Fukuda, Yumi Kotoda, Kenji Kashiwagi

**Affiliations:** Department of Ophthalmology, Faculty of Medicine, University of Yamanashi, Shimokato 1110, Chuo 409-3821, Japan; smisa@yamanashi.ac.jp (M.K.); ysugiyama@yamanashi.ac.jp (Y.F.); ykanai@yamanashi.ac.jp (Y.K.); kenjik@yamanashi.ac.jp (K.K.)

**Keywords:** polypoidal choroidal vasculopathy, complete regression of polyp, retreatment, VEGF inhibitor, number of additional injections

## Abstract

**Purpose:** This study aimed to evaluate how the temporal dynamics of polyp regression influence 24-month outcomes in eyes with polypoidal choroidal vasculopathy (PCV) treated with as-needed vascular endothelial growth factor (VEGF) inhibitors. **Methods:** Sixty-three eyes of 63 patients were included. All patients initially received three monthly intravitreal injections of either aflibercept (2 mg) or brolucizumab, followed by as-needed treatment for 24 months. The selection of a VEGF inhibitor depends on time. Indocyanine green angiography (ICGA) was performed at baseline, 3 months, and 12 months in all patients. **Results:** Among the 63 eyes, complete regression was observed in 39 eyes (61.9%) at 3 months. Of these 39 eyes, 29 eyes (46.0%) maintained complete regression at 12 months. Among the 24 eyes that did not show complete regression at 3 months, 5 eyes (20.8%) achieved complete regression at 12 months. Visual acuity improved over 24 months regardless of the timing of regression. Early regression at 3 months was associated with fewer additional injections during the first year. In contrast, regression at 12 months—rather than at 3 months—was more strongly associated with fewer additional injections during the second year. **Conclusions:** The most recent status of the polypoidal lesion was associated with the number of additional injections during the corresponding 12-month period, suggesting that ICGA-based assessment may be important for predicting treatment responsiveness.

## 1. Introduction

Polypoidal choroidal vasculopathy (PCV) is a specific subtype of neovascular age-related macular degeneration (nAMD), particularly prevalent in Asian populations [1], and is characterized by branching vascular networks and aneurysmal polypoidal lesions [2,3]. Although anti–vascular endothelial growth factor (VEGF) therapy has become the standard treatment for PCV, the clinical course of the disease remains heterogeneous, and long-term visual outcomes vary widely among patients. One of the key determinants of treatment response is the regression or persistence of polypoidal lesions, which can be evaluated using indocyanine green angiography (ICGA). Early polyp closure has been associated with favorable anatomical stabilization, reduced exudative activity, and decreased treatment burden [4,5]; however, the extent to which early angiographic outcomes predict long-term visual prognosis remains incompletely understood.

Previous studies have demonstrated that combination therapy with photodynamic therapy (PDT) and anti-VEGF agents can achieve higher rates of early polyp regression compared with anti-VEGF monotherapy [6]. Nevertheless, the clinical relevance of polyp closure at specific time points—such as 3 months or 12 months—has not been fully clarified in the context of contemporary anti-VEGF treatment strategies. In particular, it remains uncertain whether early angiographic success translates into sustained functional benefits over multiple years or whether late or incomplete polyp regression may still allow for acceptable long-term outcomes under consistent treatment.

In recent years, the introduction of newer anti-VEGF agents with enhanced durability and stronger drying effects, such as brolucizumab, has further reshaped treatment paradigms for PCV [7,8,9]. Brolucizumab, however, is known to carry a risk of intraocular inflammation, and serious complications such as retinal vasculitis and retinal vascular occlusion have been reported. In Japanese populations, the incidence of intraocular inflammation has been reported to be approximately 10%, whereas the incidence of retinal vasculitis or vascular occlusion is around 3.9% [10,11]. These agents may influence the temporal pattern of lesion regression, potentially altering the prognostic value of early ICGA findings. Moreover, real-world clinical practice increasingly incorporates individualized treatment regimens, including pro re nata (PRN), which may modulate the relationship between early anatomical responses and subsequent visual trajectories [12,13]. Despite these advances, robust evidence linking the timing of polyp regression with long-term functional outcomes remains limited, particularly in cohorts treated exclusively with anti-VEGF monotherapy.

Given the chronic and recurrent nature of PCV, identifying early biomarkers that can reliably predict long-term visual stability is of substantial clinical importance. Understanding the prognostic value of polyp closure at different stages of therapy may help refine treatment algorithms, optimize follow-up intervals, and guide personalized therapeutic decisions. By evaluating both early angiographic responses and long-term functional outcomes, this study aims to clarify the temporal relationship between lesion regression at 3 and 12 months and retreatment burden, as well as its association with visual prognosis, thereby contributing to improved management strategies for PCV.

## 2. Results

**Figure 1:**
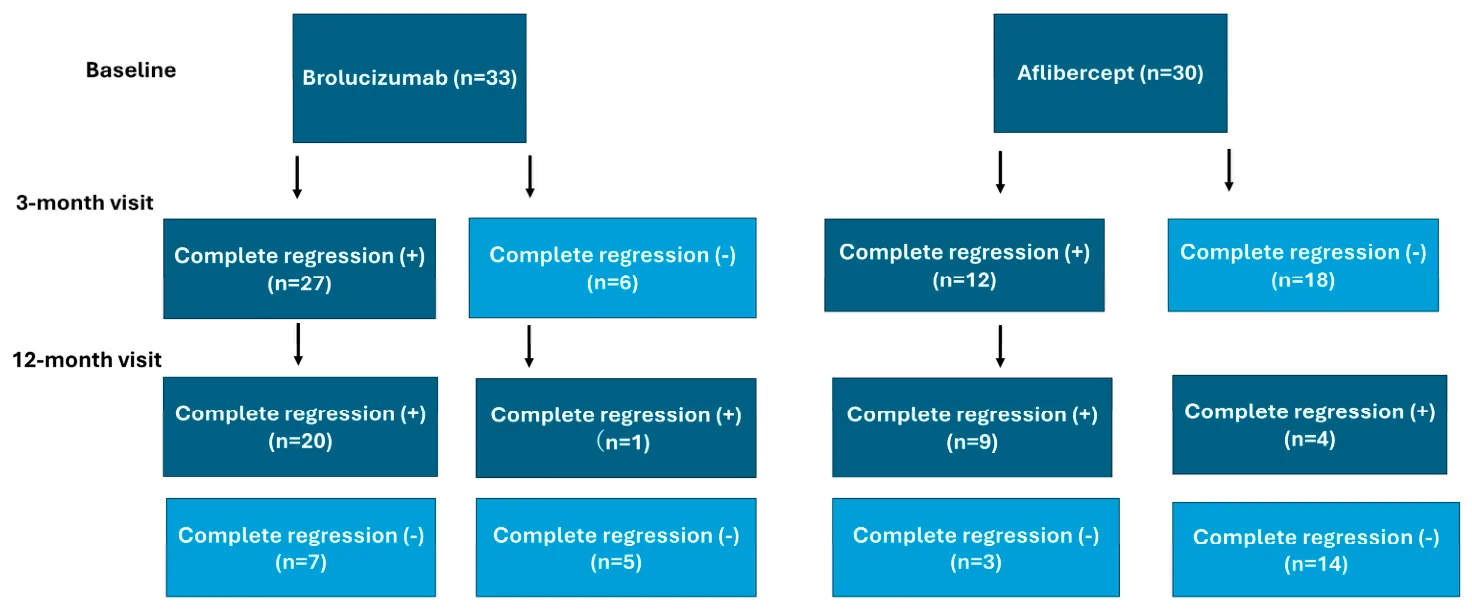
The temporal changes in complete regression on indocyanine green angiography in eyes with polypoidal choroidal vasculopathy treated with brolucizumab or aflibercept. In eyes treated with brolucizumab, the complete regression of polyps was 81.8% and 63.6% at 3 and 12 months, respectively. In eyes treated with aflibercept, the complete regression of polyps was 40% and 43.3% at 3 and 12 months, respectively.

During the study period, a total of 63 patients met the inclusion criteria and were included in the analysis. Thirty eyes were treated with aflibercept and 33 eyes with brolucizumab(Novartis Pharma Stein AG, Stein, Switzerland). This study is an extension of our previously reported 2024 study. Among the 63 eyes included in the present analysis, 60 were also part of the earlier cohort. Table 1 summarizes the baseline demographic and clinical characteristics of patients with PCV. Table 2 presents the comparison between patients with and without polyp regression at 3 months. Patients who achieved complete regression at 3 months had significantly smaller baseline polyp size and were more likely to have been treated with brolucizumab. Figure 1 shows the temporal changes in the complete regression of polypoidal lesions with the two drugs.

**Figure 2:**
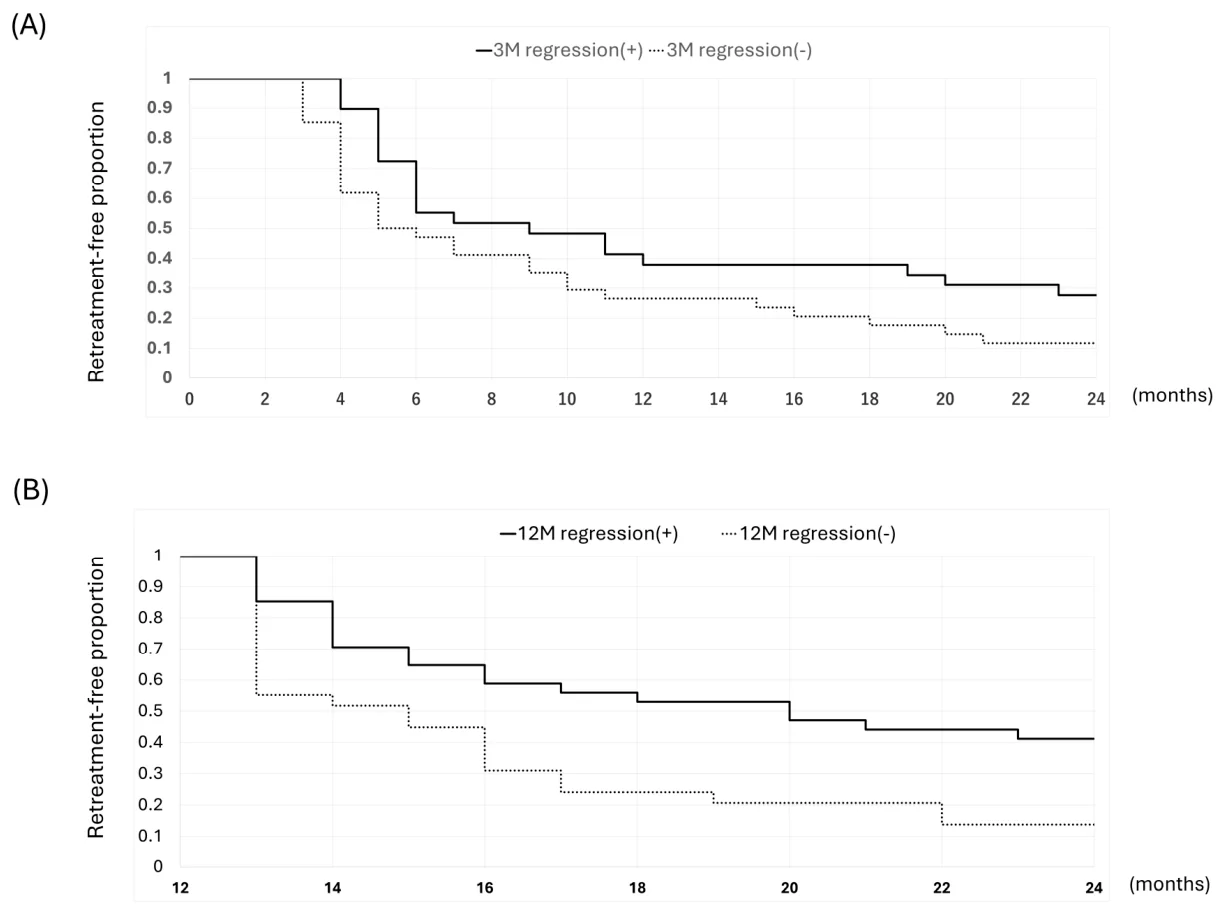
Retreatment-free proportion in eyes with polypoidal choroidal vasculopathy, stratified by complete regression at 3 or 12 months: (**A**) In eyes with or without complete regression at 3 months, the retreatment-free proportion during the 24-month study period was 25.6% and 8.3%, respectively (*p* = 0.010, log-rank test). (**B**) In eyes with or without complete regression at 12 months, the retreatment-free proportion during the second 12 months was 41.2% and 13.8%, respectively (*p* = 0.008, log-rank test).

During the first 12 months, eyes that showed polyp regression at 3 months required significantly fewer additional injections (1.3 ± 1.4 vs. 3.5 ± 3.0, *p* = 0.004 Mann–Whitney U test) and had a longer mean time not requiring the first additional injection compared with those without regression (8.7 ± 3.2 months vs. 6.4 ± 3.7 months, *p* = 0.004, Mann–Whitney U test). Figure 2 shows the Kaplan–Meier survival curve for the retreatment-free proportion over 24 months, stratified by the presence or absence of complete polyp regression at 3 months, and the retreatment-free proportion during the second 12-month period, stratified by the presence or absence of complete polyp regression at 12 months. During the 24-month study period, the proportion of eyes not requiring additional injections was higher in those with complete regression at 3 months than in those without (25.6% vs. 8.3%, *p* = 0.010, log-rank test). In the second 12-month period, the retreatment-free proportion was 41.2% in eyes with complete regression at 12 months and 13.8% in those with incomplete regression (*p* = 0.008, log-rank test).

Cataract surgery was performed in four eyes during the second 12-month period. Of these, two eyes showed complete regression at both 3 and 12 months, one eye showed complete regression at 3 months but not at 12 months, and one eye showed complete regression at 12 months but not at 3 months.

**Figure 3:**
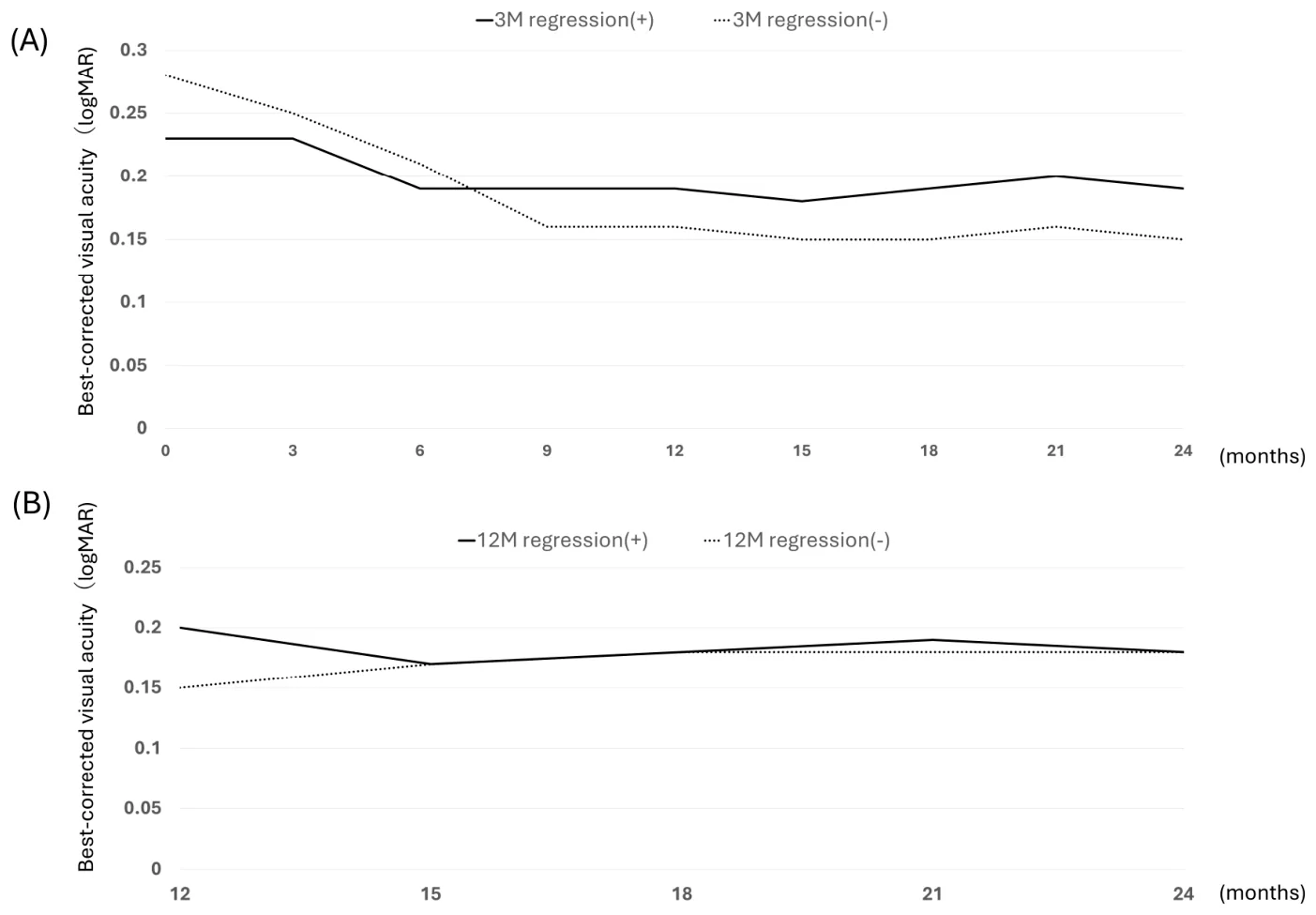
Change in best-corrected visual acuity in eyes with PCV, stratified by complete regression at 3 or 12 months: (**A**) In eyes with complete regression at 3 months, best-corrected visual acuity (BCVA) improved from 0.23 ± 0.29 to 0.19 ± 0.29 during the 24-month study period; however, the change was not statistically significant (*p* = 0.18, paired *t*-test). In eyes without complete regression at 3 months, BCVA significantly improved from 0.28 ± 0.28 to 0.15 ± 0.22 (*p* = 0.024, paired *t*-test). The intergroup difference in BCVA improvement was not significant (*p* = 0.22, Mann–Whitney U test). (**B**) In eyes with complete regression at 12 months, BCVA improved from 0.20 ± 0.28 to 0.18 ± 0.28 during months 12–24; however, the change was not statistically significant (*p* = 0.31, paired *t*-test). In eyes without complete regression at 12 months, BCVA declined from 0.15 ± 0.27 to 0.18 ± 0.25, but this change was also not statistically significant (*p* = 0.24, paired *t*-test). The intergroup difference in BCVA change was statistically significant (*p* = 0.046, Mann–Whitney U test).

Table 3 presents the comparison between patients with and without polyp regression at 12 months. No baseline factors were associated with complete regression at 12 months. Table 4 shows the Poisson regression analysis for the number of additional injections during the first 12 months. In this analysis, complete polyp regression at 3 months (*p* = 1.0 × 10^−6^), brolucizumab (*p* = 0.01), and younger age (*p* = 0.009) were factors associated with a reduced number of additional injections. Table 5 shows the Poisson regression analysis for the number of additional injections during the second 12-month period. Complete regression at 12 months (*p* = 3.3 × 10^−6^) and brolucizumab (*p* = 8.8 × 10^−4^) were associated with a reduced number of additional injections during the second 12 months. Figure 3 illustrates the 24-month change in BCVA stratified by complete polyp regression at 3 months, and the change in BCVA during months 12–24 stratified by complete polyp regression at 12 months. Table 6 shows the anatomical outcomes after treatments. Throughout the 24-month study period, the rate of dry macula remained in the 60% range. In addition, the incidence of macular atrophy was 4.8% at 24 months.

## 3. Discussion

In this retrospective study, we examined how the temporal dynamics of polyp regression influence 24-month outcomes in eyes with polypoidal choroidal vasculopathy (PCV) treated with an as-needed regimen of VEGF inhibitors. By performing ICGA at baseline, 3 months, and 12 months, we characterized the evolution of polypoidal lesions and assessed how early and later regression patterns relate to treatment burden and visual outcomes. Our findings emphasize the value of ICGA-based monitoring and suggest that the most recent status of polypoidal lesions may be a key determinant of subsequent treatment responsiveness. In this study, patients were followed under a PRN regimen for 24 months, requiring an average of 6.7 additional injections in the aflibercept group and 3.5 in the brolucizumab group. In a previous report in which PCV was treated with a treat-and-extend regimen using 2 mg aflibercept for 2 years [14], the number of additional injections was 9.3, indicating that proactive treatment regimens tend to require more injections compared with our study. Regarding visual outcomes, that report showed an improvement in visual acuity from 0.27 to 0.15 (log MAR) at 24 months, and in our study, both baseline visual acuity and the degree of improvement at 24 months were comparable, with no notable differences from previous findings.

Despite these temporal changes in polyp status, best-corrected visual acuity (BCVA) improved over 24 months regardless of whether complete regression was achieved at 3 or 12 months; however, in eyes that achieved complete regression at 3 months, BCVA improvement was not statistically significant. This suggests that visual outcomes in PCV are not determined solely by polyp regression but rather by the overall control of exudation and preservation of macular integrity. VEGF inhibitor therapy effectively reduces subretinal and intraretinal fluid, and our findings indicate that functional improvement can occur even when polyps persist. This is consistent with prior reports showing that visual acuity in PCV is influenced by multiple factors—including integrity of the ellipsoid zone, choroidal thickness, and hemorrhage—beyond the presence of polyps alone.

However, the timing of polyp regression influenced treatment burden. Eyes with complete regression at 3 months required fewer additional injections during the first 12 months, suggesting that early responders may have a more favorable disease phenotype or a stronger biological response to VEGF inhibition. In contrast, complete regression at 12 months was associated with fewer injections during the second 12-month period. The number of additional injections in the second 12-month period was associated with brolucizumab, along with complete regression at 12 months. This finding suggests that later regression still confers clinical benefit by reducing long-term treatment burden. The association with brolucizumab is noteworthy, as this agent has been reported to induce higher rates of fluid resolution and may exert a stronger effect on polypoidal lesions compared with other anti-VEGF agents [15,16]. Although our study was not designed to compare agents directly, the observed association suggests that drug selection may influence polyp regression and subsequent treatment requirements. Importantly, the number of additional injections during each 12-month period was associated with the most recent polyp status, underscoring the importance of ICGA-based assessment in PCV management. While OCT and OCTA are widely used for routine monitoring, ICGA remains the gold standard for visualizing polypoidal lesions. Our findings suggest that ICGA provides meaningful prognostic information that may help clinicians anticipate treatment burden and tailor follow-up strategies. Identifying persistent or recurrent polyps may prompt closer monitoring or consideration of alternative therapeutic approaches.

These results also raise questions about the optimal timing and frequency of ICGA in clinical practice. Although ICGA is not routinely performed at fixed intervals, our findings indicate that periodic ICGA assessment may provide additional prognostic information. Future prospective studies are needed to determine whether ICGA-guided treatment strategies can improve long-term outcomes or reduce overtreatment.

This study has several limitations. Its retrospective design introduces potential selection bias, and although the sample size is reasonable for a single-center study, the generalizability of the findings is limited. The choice of VEGF inhibitor drugs varied over time, reflecting real-world practice rather than randomized allocation. Although we observed an association between brolucizumab use and 12-month regression, this should be interpreted with caution, as temporal trends or unmeasured confounders may influence the association. Additionally, we did not evaluate other potential predictors of treatment response, such as branching vascular network morphology or genetic factors [17,18], which may also influence polyp dynamics. Furthermore, the fact that six patients were lost to follow-up during the 24-month observation period may also introduce potential study bias. Despite these limitations, our study provides new insights into the temporal behavior of polypoidal lesions and their relationship to treatment burden in PCV. The results suggest that early and late polyp regression have distinct clinical implications and that ICGA-based assessment plays an important role in understanding disease activity. Importantly, visual improvement was achieved regardless of polyp regression status, highlighting the effectiveness of VEGF inhibitor therapy in maintaining macular function over the long term; however, BCVA improvement was not statistically significant in eyes achieving complete regression at 3 months. This might be due to good baseline BCVA and a small sample size. ICGA is an invasive examination, and in rare cases, it can cause severe adverse reactions such as anaphylactic shock. Although the current detection capability of OCTA for polypoidal lesions remains limited [19,20,21], future improvements in imaging performance may allow clinicians to assess the presence or absence of polypoidal lesions using OCTA, a far less invasive approach.

In conclusion, the temporal dynamics of polyp regression are closely linked to treatment burden in PCV, with the most recent polyp status serving as a meaningful indicator of future injection requirements. These findings support the value of ICGA as a marker of treatment burden and suggest that individualized treatment strategies based on polyp behavior may help optimize long-term outcomes.

## 4. Methods

This retrospective study included consecutive patients with treatment-naïve PCV who met the following inclusion criteria: (1) treatment-naïve eyes with PCV, and (2) initial treatment consisting of three monthly injections of aflibercept (2 mg) (Bayer AG, Leverkusen, Germany) or brolucizumab, followed by as-needed injections over 24 months. The choice of VEGF inhibitor was time-dependent: aflibercept (2 mg) was administered between January 2015 and June 2020, whereas brolucizumab was used between July 2020 and December 2023. The exclusion criteria were (1) exudative maculopathy other than PCV and (2) prior or concurrent treatments other than VEGF inhibitors, including photodynamic therapy (PDT) or combination therapy with PDT and a VEGF inhibitor. This retrospective study was approved by the Institutional Review Board of the University of Yamanashi (approved No. 3025) and performed according to the tenets of the Declaration of Helsinki (approved No. 2892). Before treatment initiation, each patient provided written informed consent.

At baseline, and at 3 and 12 months, all patients underwent a comprehensive ophthalmic examination, including measurement of best-corrected visual acuity (BCVA) using the Landolt chart, intraocular pressure assessment, slit-lamp biomicroscopy with or without a 78-diopter lens, color fundus photography, spectral-domain optical coherence tomography (SD-OCT; Spectralis HRA2, Heidelberg, Dossenheim, Germany), swept-source OCT (SS-OCT; DRI-1 OCT, Topcon, Japan), and fluorescein/indocyanine green angiography (FA/ICGA) using a confocal scanning ophthalmoscope (Spectralis HRA2). At all other visits, patients received the same comprehensive examination except for FA/ICGA. 

All patients received three consecutive monthly injections of either aflibercept (2.0 mg/0.05 mL) or brolucizumab (6.0 mg/0.05 mL), followed by monthly follow-up visits and additional injections on an as-needed basis for up to 24 months. The choice of VEGF inhibitor was determined by the treatment period: aflibercept was administered before July 2020, whereas brolucizumab was used thereafter. Additional injections were given when OCT revealed subretinal or intraretinal fluid or when a new subretinal hemorrhage was detected by ophthalmoscopy. An increase in pigment epithelial detachment was not considered an indication for additional treatment in this study. The OCT scan protocol consisted of a cross-hair pattern with a 9 mm scan length centered on the fovea. The presence or absence of complete polyp regression was assessed on ICGA by two independent graders (M.K. and Y.F.). In cases of disagreement, the final decision was made by a senior third grader (Y.S.).

Statistical analyses were performed using SPSS ver.12 (IBM, Tokyo, Japan). The chi-square test and the Mann–Whitney U test were used to evaluate differences in categorical and continuous variables, respectively. Factors associated with the number of additional injections were analyzed using Poisson regression. Differences between baseline and post-treatment values were assessed using a paired *t*-test. A log-rank test was conducted to compare the time from baseline to the first additional injection between the two groups. Statistical significance was defined as *p* < 0.05.

## Figures and Tables

**Table 1:** Baseline characteristics in patients with polypoidal choroidal vasculopathy.

Age (Years)	72.6 ± 7.9
Male/Female	47/16
Best-corrected visual acuity (log MAR)	0.25 ± 0.28
Central retinal thickness (μm)	331 ± 143
Subfoveal choroidal thickness (μm)	209 ± 87
Number of polyp(s)	2.9 ± 2.6
Maximum diameter of polyp	361 ± 210
VEGF inhibitor(aflibercept/brolucizumab)	30/33

**Table 2:** Comparison of baseline characteristics between patients with or without polyp regression at 3 months.

	Regression (+) (n = 39)	Regression (−) (n = 24)	*p*-Value
Age (years)	73.1 ± 7.6	71.7 ± 8.5	0.38
Male/Female	30/9	17/7	0.59
BCVA (log MAR)	0.23 ± 0.29	0.28 ± 0.28	0.46
Central retinal thickness (μm)	332 ± 154	327 ± 125	0.77
Subfoveal choroidal thickness (μm)	210 ± 94	206 ± 74	1.00
Number of polyp(s)	2.6 ± 2.5	3.3 ± 2.8	0.079
Maximum diameter of polyp	307 ± 162	448 ± 251	0.036
VEGF inhibitor (aflibercept/brolucizumab)	12/27	18/6	0.001

BCVA: best-corrected visual acuity; log MAR: logarithm of minimum angle resolution; VEGF: vascular endothelial growth factor.

**Table 3:** Comparison of baseline characteristics between patients with or without polyp regression at 12 months.

	Regression (+) (n = 34)	Regression (−) (n = 29)	*p*-Value
Age (years)	71.2 ± 7.1	73.7 ± 8.8	0.34
Male/Female	25/9	22/7	0.83
BCVA (log MAR)	0.24 ± 0.28	0.27 ± 0.29	0.80
Central retinal thickness (μm)	335 ± 154	324 ± 131	0.71
Subfoveal choroidal thickness (μm)	207 ± 92	210 ± 82	0.67
Number of polyp(s)	2.6 ± 2.7	3.2 ± 2.6	0.071
Maximum diameter of polyp	320.0 ± 187	409 ± 228	0.10
VEGF inhibitor (aflibercept/brolucizumab)	13/21	17/12	0.11

BCVA: best-corrected visual acuity; log MAR: logarithm of minimum angle resolution; VEGF: vascular endothelial growth factor.

**Table 4:** Factors associated with the number of additional injections during the first 12-month period.

	B	SE	Waldχ^2^	*p*-Value	Exp (B)
Age (year)	0.029	0.011	6.8	0.009	0.007 to 0.051
Treatment (1. Brolucizumab, 0: aflibercept)	0.52	0.20	6.6	0.01	0.12 to 0.92
Regression of polyp(s) at 3 months	0.79	0.20	23.8	1.1 × 10^−6^	0.58 to 0.98

Baseline best-corrected visual acuity, central retinal thickness, subfoveal choroidal thickness, the number of polyps, and maximum polyp size were eliminated in this analysis.

**Table 5:** Factors associated with the number of additional injections during the second 12-month period.

	B	SE	Waldχ^2^	*p*-Value	Exp (B)
Treatment (1. Brolucizumab, 0: aflibercept)	0.55	0.17	11.1	8.8 × 10^−4^	0.23 to 0.87
Regression of polyp(s) at 12 months	0.78	0.17	21.6	3.3 × 10^−6^	0.55 to 0.98

Age, sex, baseline best-corrected visual acuity, central retinal thickness, subfoveal choroidal thickness, the number of polyps, regression of polyp(s) at 3 months, and maximum polyp size were eliminated in this analysis.

**Table 6:** Morphological outcomes after treatments.

	Baseline	6 M	12 M	18 M	24 M
CRT(μm)	330 ± 143	231 ± 93	231 ± 125	237 ± 89	241 ± 79
SRF (%)	90.5	25.4	19.1	25.4	30.2
IRF (%)	11.1	6.3	4.8	6.4	7.9
Dry macula rate(%)	0	70	78	70	65
sPED (%)	54.0	22.2	22.2	20.6	22.2
fPED (%)	4.8	9.5	11.1	9.5	9.5
MA (%)	1.6	3.2	3.2	4.8	4.8

CRT: central retinal thickness; SRF: subretinal fluid; IRF: intraretinal fluid; sPED: serous pigment epithelial detachment; fPED: fibrovascular pigment epithelial detachment; MA: macular atrophy.

## Data Availability

The data presented in this study are available upon request from the corresponding author due to privacy restrictions.
